# Pereira’s Materia Medica

**Published:** 1843-12

**Authors:** 


### PEREIRA'S MATERIA MEDICA.

A review of this very valuable work will appear in our next number.

